# Remnant Woven Bone and Calcified Cartilage in Mouse Bone: Differences between Ages/Sex and Effects on Bone Strength

**DOI:** 10.1371/journal.pone.0166476

**Published:** 2016-11-09

**Authors:** Victoria Ip, Zacharie Toth, John Chibnall, Sarah McBride-Gagyi

**Affiliations:** 1 School of Operations Research and Information Engineering, Cornell University, Ithaca, New York, United States of America; 2 Department of Orthopaedic Surgery, Saint Louis University, Saint Louis, Missouri, United States of America; 3 Department of Psychiatry, Saint Louis University, Saint Louis, Missouri, United States of America; Indiana University Purdue University at Indianapolis, UNITED STATES

## Abstract

**Introduction:**

Mouse models are used frequently to study effects of bone diseases and genetic determinates of bone strength. Murine bones have an intracortical band of woven bone that is not present in human bones. This band is not obvious under brightfield imaging and not typically analyzed. Due to the band’s morphology and location it has been theorized to be remnant bone from early in life. Furthermore, lamellar and woven bone are well known to have differing mechanical strengths. The purpose of this study was to determine (i) if the band is from early life and (ii) if the woven bone or calcified cartilage contained within the band affect whole bone strength.

**Woven Bone Origin Studies:**

In twelve to fourteen week old mice, doxycycline was used to label bone formed prior to 3 weeks old. Doxycycline labeling and woven bone patterns on contralateral femora matched well and encompassed an almost identical cross-sectional area. Also, we highlight for the first time in mice the presence of calcified cartilage exclusively within the band. However, calcified cartilage could not be identified on high resolution cone-beam microCT scans when examined visually or by thresholding methods.

**Mechanical Strength Studies:**

Subsequently, three-point bending was used to analyze the effects of woven bone and calcified cartilage on whole bone mechanics in a cohort of male and female six and 13 week old Balb/C mice. Three-point bending outcomes were correlated with structural and compositional measures using multivariate linear regression. Woven bone composed a higher percent of young bones than older bones. However, calcified cartilage in older bones was twice that of younger bones, which was similar when normalized by area. Area and/or tissue mineral density accounted for >75% of variation for most strength outcomes. Percent calcified cartilage added significant predictive power to maximal force and bending stress. Calcified cartilage and woven bone could have more influence in genetic models where calcified cartilage percent is double our highest value.

## Introduction

**Fig 1:**
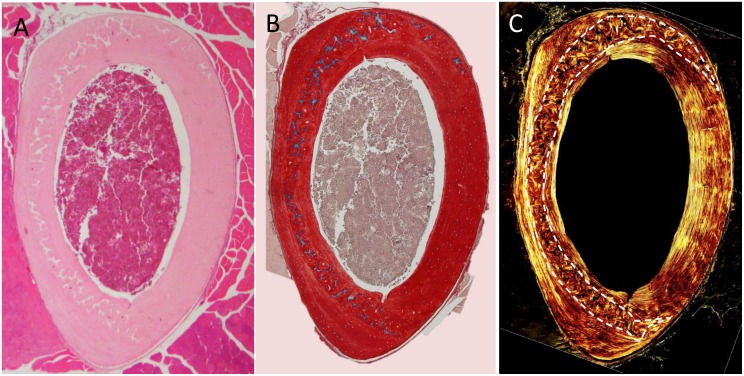
Comparison of ICWB visualization with standard histological stains and brightfield imaging versus polarized imaging. Two serial sections from the mid-shaft of a 13-week-old male mouse were stained with either H&E (A) or picrosirius red and alcian blue (B & C). (A) Under brightfield imaging neither the ICWB nor calcified cartilage is easily differentiated from surrounding lamellar bone using an H&E stain. (B) Alcian blue staining allows visualization of calcified cartilage, but the ICWB is still not obvious. (C) Only under polarized light imaging can the ICWB (white dashed lines) be differentiated.

Mouse models are used frequently to study effects of bone diseases and genetic determinates of bone strength. Multiple factors affect whole bone strength and have been extensively studied over the past decades. A few of the well-studied and often analyzed are size/cross sectional area, bone mineral density (BMD), and collagen alignment. Like any object, bones with larger cross-sectional area are stronger than those of the same material with smaller cross-sectional area. Decreased BMD, which occurs in disease states like osteoporosis, may indicate a change in the bone material itself and an overall decrease in bone strength while whole bone geometry is unaffected [1,2]. The correlations between BMD and whole bone strength are so strong that BMD measurements via dual emission x-ray absorptiometry (DEXA) are currently the most widely used clinical determinate of future fracture risk [3]. Collagen fibrils can assemble into lamellar bone, a well-organized and aligned bone matrix, or woven bone, which is disorganized and non-aligned. Mechanically, lamellar bone is anisotropic but very strong in its primary loading direction; woven bone is more isotropic but much weaker in any loading direction. The importance of proper collagen alignment in bone is well demonstrated by Paget’s disease of bone. In Paget’s disease osteoclast activity is markedly increased at localized sites within the body. The increased resorption accelerates bone formation by osteoblasts to replace the removed tissue. However, the new tissue is not well organized and is a hypomineralized mosaic of lamellar and woven bone. Consequently, the tissue is more compliant and less hard [4]. While all three sub-macro level factors (size, BMD, and collagen alignment) have been well studied, only size and BMD or tissue mineral density (TMD) are routinely examined in rodent-based biomechanical phenotyping experiments [5]. It should be noted that several other measurement techniques (e.g. Raman spectroscopy, Fourier Transform Infrared spectroscopy, X-ray Diffraction) have been used to examine lower length-scale tissue features such as matrix crystalline composition, mineral to matrix ratio, and matrix cross-linking. For the purposes of this study, sub-macro level architecture was selected as the focus because these are features which can easily be measured with microCT or standard laboratory equipment such as a microscope. Overlooking collagen alignment is concerning because there exists an intracortical band of woven bone (ICWB) present in normal rodent bone but not in normal human long bones [6–11]. The ICWB is not typically analyzed. This is probably because the ICWB is not obvious in brightfield imaging (Fig 1A and 1B). Polarized light imaging is needed (Fig 1C).

The ICWB’s size and structure has prompted assumptions that it is unremodeled endochondral bone from early postnatal growth [6,7]. MicroCT analysis of mouse bones have shown that long bone architecture changes dramatically from E18.5 through the first 4 weeks of life [8,9]. Cortical area more than doubles over this time and maintains a highly porous structure which is presumably entirely woven bone. Only after 4 weeks of age does growth slow and bones appear compact on microCT [8,9]. It is plausible that during the later, slower phases of growth lamellar bone is laid over the woven bone core thus creating the ICWB structure. However, this has not been definitively proven.

Another finding that supports the remnant bone concept of the ICWB’s origins is the presence of calcified cartilage exclusively within the ICWB [6,7]. It is presumed that the calcified cartilage is a remnant from endochondral bone formation at the growth plate. To date, studies where calcified cartilage in diaphyseal bone is the main focus are few and have only been done on rats [6,7]. Calcified cartilage was found to be hypermineralized and stiffer than surrounding bone, but whole bone mechanical properties were not analyzed [6,7]. It is not clear if increased/decreased presence of calcified cartilage would affect whole bone strength. Furthermore, the presence of calcified cartilage in mouse cortical bone has not been a main focus of any literature to date.

Based on the available information about rodent long bone growth in addition to ICWB and calcified cartilage in rats we hypothesize that the ICWB in mice is unremodeled bone from early life and that the amount of woven bone and calcified cartilage present can affect bone strength.

The objectives of this study were to (i) verify that mice have an intracortical band of woven bone that contains calcified cartilage islands similar to rats, (ii) determine if the intracortical band of woven bone found in mice is unremodeled endochondral bone formed early in growth, and (iii) ascertain if either the amount of woven bone or calcified cartilage has an effect on whole bone mechanics. Indeed, it was found that this woven bone is from early postnatal growth and contains calcified cartilage islands. Also, it was found that both calcified cartilage and woven bone can impact whole bone strength albeit with significantly less influence than TMD or size.

## Materials and Methods

### Animal Ethics Statement

All procedures were carried out with strict accordance to national animal welfare guidelines and with the approval of Washington University in St. Louis’s and/or Saint Louis University’s IACUC committee (protocols 20110209 and 2382, respectively). Animals were group housed under standard husbandry conditions and given access to food and water *ad libitum*. Daily care was performed by comparative medicine’s husbandry staff. All animals were euthanized by gradual carbon dioxide asphyxia according to the American Veterinary Medical Association’s guidelines.

### Woven Bone Origin Studies

#### Animals

Twelve to 14 week old male (n = 4) and female (n = 1) mice generated as control calibration mice for a previous study were used (*Bmp*^*fl/fl*^ on a C57BL/6 background) [12]. Doxycycline (DOX), a fluorescent tetracycline, was administered *in utero* to 3 weeks old via drinking water to the pregnant mother and the animal directly (1 μg/mL in 3–5% sugar water). DOX was given to all animals in the previous study to repress Cre expression in knockout littermates (*Bmp2*^*fl/fl*^*; OSX-Cre)*, but DOX also incorporates into any actively mineralizing tissues [13]. So, all bone formed prior to 3-weeks-old was fluorescently labeled. After euthanasia, both femora were harvested, fixed overnight in 10% neutral buffered formalin, and scanned with high resolution cone-beam microCT (uCT 35, Scanco Medical, Wayne, PA; X-ray tube potential 70 kVp, integration time 800 ms, X-ray intensity114 μA, isotropic voxel size 3.5 um, frame averaging 1, 1000 projections, high resolution scan) prior to histological processing.

#### Histology

Right femora were used to determine the amount and distribution of woven bone and calcified cartilage at mid-shaft for each animal. Briefly, the bones were decalcified with EDTA, embedded in paraffin, and cross-sectioned at the midpoint. One section per animal was stained with alcian blue (Electron Microscopy Sciences, Hatfield, PA) and picrosirius red (Sigma, St. Louis, MO) to stain cartilage and bone, respectively. Picrosirius red also adds birefringence to collagen fibrils allowing better visualization of collagen alignment under polarized light [14]. Brightfield and polarized microscopic images were obtained (BX51P with camera DP70, Olympus, Waltham, MA) and analyzed using ImageJ (NIH, Bethesda, MD). Polarized images were used to measure woven bone area (WB.A). Brightfield images were used to measure cortical area (Ct.A) and calcified cartilage area (CC.A). Calcified cartilage area was identified by color thresholding for blue. Both images were compared side-by-side to determine calcified cartilage location (lamellar vs. woven bone).

**Fig 2:**
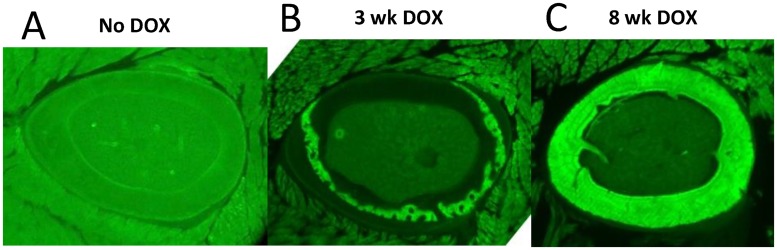
Example of DOX bone labeling in 12-14wk old mice with different administration times. Doxycycline (DOX) is a fluorescent tetracycline that can be administered to animals via drinking water and incorporates into actively mineralizing tissues. (A) Animals never exposed to DOX show no fluorescence even when imaged with a lengthy exposure (1000ms, no gain). (B) Animals exposed to DOX *in utero* until 3 weeks old have an intracortical band of labeled bone (exposure 500ms, no gain). (C) If DOX was administered until 8 weeks old, almost the entirety of the bone is labeled (exposure 500ms, no gain).

Left femora were used to determine the amount and distribution of remnant bone from early life (conception to 3-weeks-old) at the mid-shaft of each animal. Briefly, femora were plastic embedded in polymethyl methacrylate (Sigma, St. Louis, MO) and cross-sectioned at the midpoint using a slow speed diamond saw (SP1600, Leica Microsystems, Buffalo Grove, IL). After polishing to 30 to 50um thickness, the plastic sections were fluorescently imaged to visualize DOX labeled bone (CTR4000 with DFC340FX camera, Leica Microsystems). Images were analyzed with ImageJ to determine Ct.A and DOX labeled bone area (remnant early bone, EB.A). To verify DOX labeling, positive (12-week-old M/F mice given DOX until 8 weeks old, n = 4) and negative (12 to 14-week-old M/F mice never exposed to DOX, n = 4) control animals were processed similarly (Fig 2).

#### Identification of Calcified Cartilage on High Resolution MicroCT

High resolution microCT scans (3.5um voxel size) were used in conjunction with histological sections to try to identify calcified cartilage in three dimensions. Two previous studies have shown that calcified cartilage is hyper mineralized compared to surrounding bone [6,7]. However, one stated that it was unlikely that cone beam microCT could be used to image them due to their irregular shape and small feature sizes. Ultimately the goal of this outcome was to determine if calcified cartilage could be quantified using cone-beam microCT, which is a common imaging modality for biomechanical phenotyping [5]. First, the microCT slice most resembling the histological section for each animal was identified manually. The two were then compared visually for similar patterns as higher mineralized areas appear whiter. A thresholding method was subsequently employed. The pass threshold was gradually increased until either the pattern matched the calcified cartilage patterns or no bone remained. If the former could be attained then it would establish a threshold that could be used to quantify calcified cartilage. On the other hand, if the pattern could not be matched in a majority of the samples, then this would indicate that cone-beam microCT cannot be used to measure calcified cartilage.

#### Statistical Analysis

Paired t-tests (Statview, SAS Institute, Cary, NC) were used to compare the calcified cartilage locations (woven vs. lamellar) as well as percent DOX labeled bone to percent WB.A. A p-value of <0.05 was considered significant. Data is presented as mean with standard deviation.

### Mechanical Strength Studies

#### Animals

A cohort of 6 week old male and female Balb/C mice were purchased (n = 10/sex, Jackson Laboratories, Bar Harbor, ME). Half were directly euthanized, and half were housed in standard conditions until euthanasia at 13-weeks-old. After euthanasia, both femora were harvested. Right femora were fixed overnight and processed for histology. Left femora were wrapped in saline soaked gauze and stored at -20°C until processing for mechanical testing.

#### Histology

Right femora were used to determine the amount and distribution of woven bone and calcified cartilage at mid-shaft for each animal. Briefly, the bones were decalcified with formic acid, embedded in paraffin, and cross-sectioned at the midpoint. Then identical staining, imaging, and analysis were performed as was done for the right femora for the woven bone origin studies.

#### Whole Bone Mechanical Testing

Left femora were used to determine strength and material properties. First, they were embedded in agarose to maintain hydration and scanned with microCT (μCT 35; X-ray tube potential 70 kVp, integration time 300 ms, X-ray intensity 114 μA, isotropic voxel size 12 um, frame averaging 1, 500 projections, medium resolution scan) to determine the cross-sectional morphometry and bone tissue mineral density (TMD) [15]. The manufacture provided calibration was used to convert per milles into mg HA/cm^3^. After scanning, the samples were rewrapped in saline soaked gauze and placed back into their sample tubes for transport to the mechanical testing room. Each bone was subjected to three-point bending (Criterion 42, MTS, Waller, TX, 4.9mm span, transverse load at anterior midpoint, displacement control 0.1mm/s). Force-displacement data was analyzed to determine stiffness, maximal force, displacement at maximal force, fracture displacement, energy to fracture, energy at maximal force, and post-maximal energy to fracture. Data normalized for cross-sectional morphometry using beam bending theory with maximal distance from the centroid was used to determine maximal bending stress and elastic modulus as previously detailed in [16].

#### Statistical Analysis

The effects of age and sex on WB.A (absolute and percent Ct.A) and CC.A (absolute, percent Ct.A, and percent WB.A) were compared using two-way ANOVA (factors: age and sex) (Statview). There were no significant interactions for any reported outcome. If a factor was significant, the relevant groups were compared with unpaired t-tests to identify significant differences. Bars indicating significant differences on graphs represent the unpaired t-test results. Then, multivariate associations of structural and compositional measures with three-point bending outcomes were examined using forward inclusion linear regression (candidate independent variables: TMD, cross-sectional area from microCT, WB.A, percent WB.A per Ct.A, CC.A, percent CC.A per Ct.A). Forward inclusion linear regression is an iterative process. One candidate independent variable is added to the model each round until there is no significant improvement in the model’s predictive capacity as determined by ANOVA testing. The independent variable added each iteration is selected by determining which one causes the greatest change in the coefficient of determination, R^2^, compared to the previous iteration’s model. All data points were pooled in order to increase the range of our independent variables. A p-value <0.05 was considered significant. Data is presented as mean with standard deviation.

## Results

### Woven Bone Origin Studies

**Fig 3:**
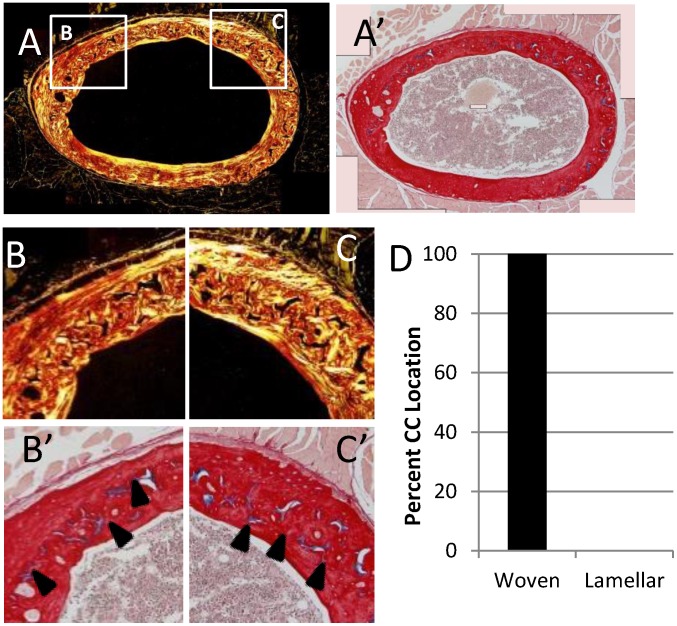
Example mouse femur histological cross section demonstrates calcified cartilage presence. (A-C) An intracortical band of woven bone (ICWB) apposed by lamellar bone was present in all samples of both studies. (A’-C’) Calcified cartilage (blue tissue, black arrow heads) were present in all samples. (D) Calcified cartilage resided exclusively within ICWB. None could be found in lamellar bone.

**Fig 4:**
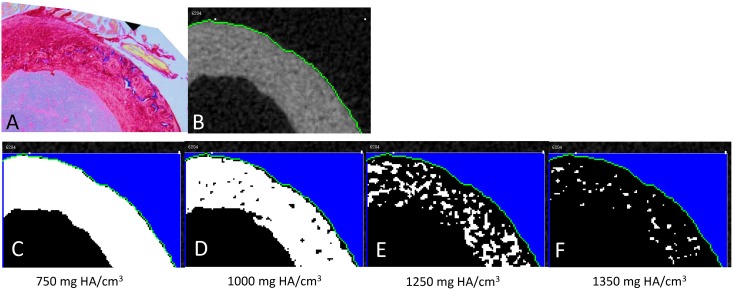
Example mouse femur histological cross section compared to high resolution microCT. (A) Calcified cartilage, which is reportedly hyper mineralized compared to surrounding bone, is easily visible in histological sections stained with alcian blue and picrosirius red (blue tissue). (B-F) However, even with high-resolution cone-beam microCT (3.5um voxel), calcified cartilage cannot be distinguished from surrounding bone via mineral density thresholding. Thus, cone-beam microCT at 3.5um resolution cannot be used to quantify calcified cartilage.

**Fig 5:**
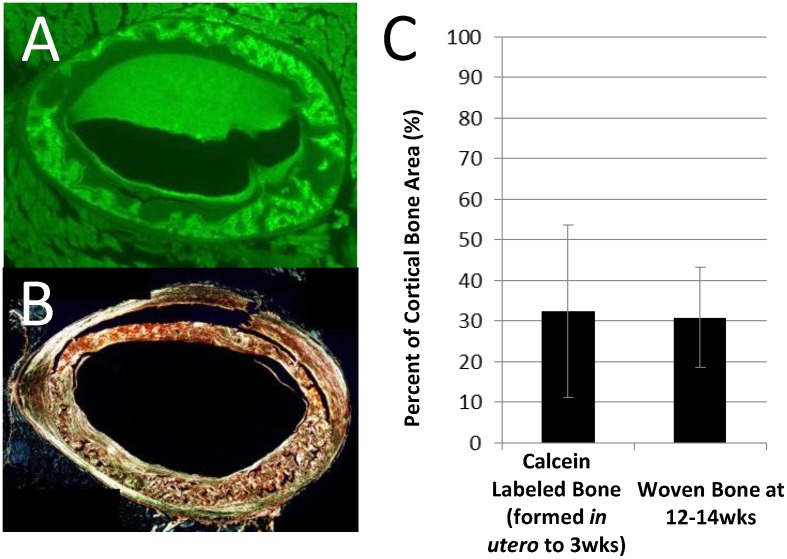
Comparison of early bone formation and ICWB in 12-14wk old mice. Bone formed up to 3 wks old (green signal, A) was similar in pattern and amount as woven bone (B) in the contralateral limb (C, n = 5).

Picrosirius red and alcian blue stained sections of the contralateral limbs were used to highlight bone and cartilage, respectively. Polarized light imaging revealed a woven bone core apposed by circumferentially-aligned lamellar bone (Fig 3A–3C). As hypothesized, alcian blue staining demonstrated the presence of calcified cartilage (Fig 3A’–3C’). Calcified cartilage was found exclusively within ICWB (Fig 3D). Calcified cartilage could not be identified via cone beam microCT for any animals (Fig 4). Doxycycline, a fluorescent tetracycline, was used to label bone formation *in utero* to 3 weeks old. At 12 to 14 weeks old all animals exposed to doxycycline exhibited an intracortical fluorescent band indicating remnant bone was present (Fig 5A). In all animals, the doxycycline-labeled areas and woven bone areas from contralateral limbs were similar in pattern and amount (Fig 5).

### Mechanical Strength Studies

#### Effects of Age and Sex on ICWB and Calcified Cartilage

**Fig 6:**
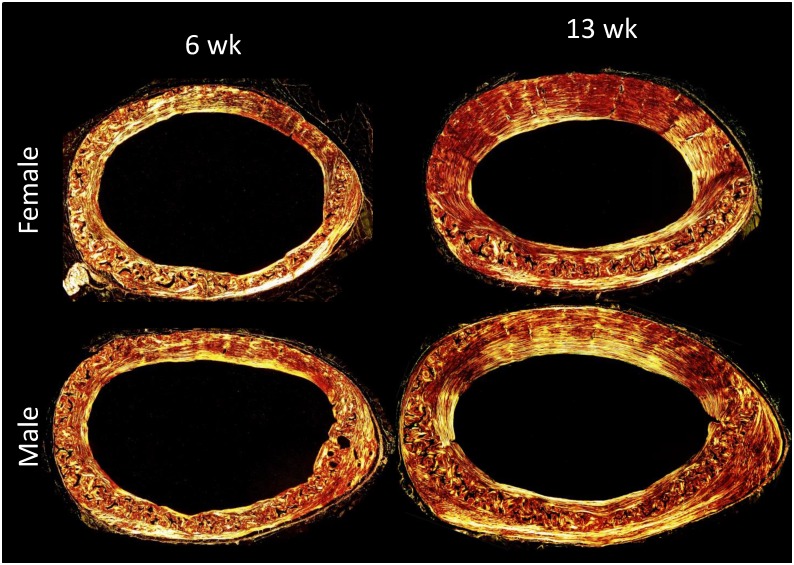
Woven Bone in 6 and 13 week old male and female Balb/C mice. Absolute woven bone area was the same between age groups of each sex with males tending to be higher than females. However, when normalized by cortical area, percent woven bone was significantly higher in the 6 week old mice than 13 week olds and there were no differences attributable to sex.

**Fig 7:**
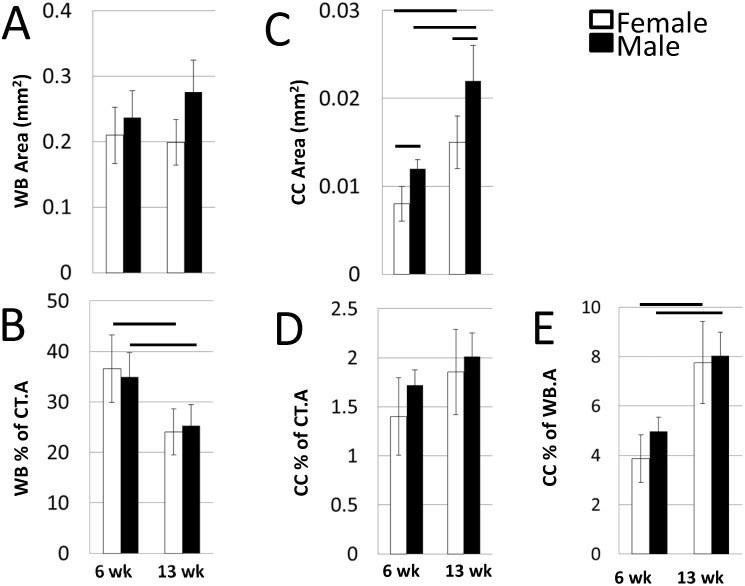
Woven Bone and Calcified Cartilage Outcomes at the Femur mid-shaft in Balb/C Mice. (A) Absolute WB.A was the same between age groups of each sex with males tending to be higher than females (ANOVA, p = 0.042). (B) However, when normalized by Ct.A, percent WB was significantly higher in the 6 week old mice than 13 week olds and there were no differences attributable to sex. (C) In contrast, the absolute amount of CC differed between ages and sex with older having twice that of younger and males having more than female. (D) When normalized by Ct.A it remained elevated in older animals but was not significant (ANOVA, p = 0.0581). (E) When normalized by WB.A, percent CC.A was significantly higher in 13week old mice than 6 week old mice.

**Fig 8:**
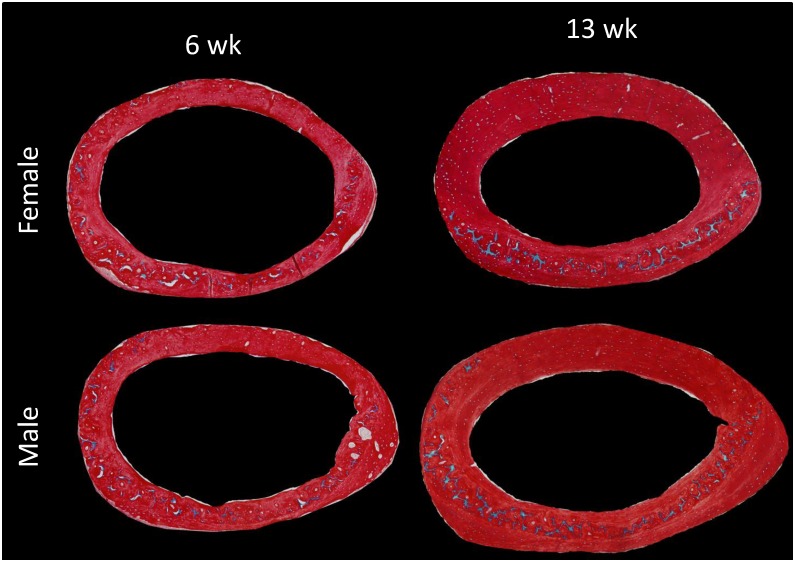
Calcified cartilage in 6 and 13 week old male and female Balb/C mice. Calcified cartilage at the femur mid-shaft was significantly higher in 13 week old animals than 6week old animals and higher in males than females. However, when normalized by cortical area the percent calcified cartilage was similar between sexes of one age but remained higher in 13week animals.

Absolute WB.A was the same between age groups of each sex; males tended to be higher than females (ANOVA, p = 0.042; unpaired t-test not significant; Figs 6 and 7A). However, when normalized by Ct.A, percent WB was significantly higher in the 6 week old mice than 13 week olds and there were no differences attributable to sex (ANOVA, p = 0.003; Fig 7B). In contrast, the absolute amount of CC differed between ages and sex with older having twice that of younger and males having more than female (ANOVA, age p <0.001, sex p = 0.003; Fig 7B and 7C). When normalized by Ct.A it remained elevated in older animals but was not significant (ANOVA, p = 0.0581; Fig 7D). When normalized by WB.A, percent CC.A was significantly higher in 13week old mice than 6 week old mice (ANOVA, p<0.001; Figs 7E and 8).

#### Effects of Woven Bone and Calcified Cartilage on Bone Strength

**Fig 9:**
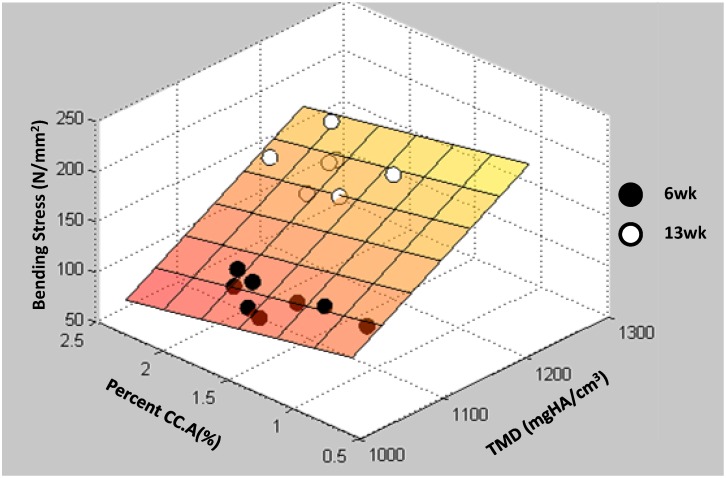
Maximal Bending Stress Correlation. As expected, TMD strongly correlated with maximal bending stress (independent R^2^ = 0.795). Inclusion of percent CC.A increased the model’s predictive capabilities bringing the final model R^2^ to 0.854.

Predictably, Ct.A and/or TMD accounted for >75% of the variation for most strength outcomes (stiffness, modulus, maximal force, bending stress, total displacement) (Table 1). Percent WB.A was the only significant factor for post-maximal force energy, and absolute WB.A was the only significant factor for total energy. However, both were weak associations (r-square value <0.30; Table 1). Percent CC.A added significant, albeit small, predictive power to maximal force (additive 2%, p < 0.05; Table 1) and bending stress (additive 6%, p < 0.05; Fig 9 & Table 1).

**Table 1:** Three point bending correlative results.

Outcome	Final Model	Final Model R^2^	R^2^ with Only the Primary Independent Variable		Increase in R^2^ with Additional Independent Variables			
**Stiffness**	0.383*TMD + 110.156*Ct.A—409.124	0.962	TMD	0.885	Ct.A	0.077	-	-
**Maximal Force**	31.104*Ct.A + 0.042*TMD -3.718*%CC.A -46.273	0.953	Ct.A	0.898	TMD	0.034	%CC.A	0.021
**Displacement at Maximal Force**	-0.001*TMD + 0.999	0.532	TMD	0.532	-	-	-	-
**Post-maximum to Fracture Displacement**	-0.002*TMD + 2.345	0.617	TMD	0.617	-	-	-	-
**Fracture Displacement**	-0.003*TMD + 3.344	0.751	TMD	0.751	-	-	-	-
**Energy to Fracture**	19.085*WB.A + 2.037	0.263	WB.A	0.263	-	-	-	-
**Energy to Maximum Force**	5.494*Ct.A -0.631	0.682	Ct.A	0.682	-	-	-	-
**Post-maximum to Fracture Energy**	0.110*%WB.A -0.767	0.266	%WB.A	0.266	-	-	-	-
**Maximal Bending Stress**	0.494*TMD—24.619*%CC.A—381.011	0.854	TMD	0.795	%CC.A	0.059	-	-
**Elastic Modulus**	15.677*TMD—24866.410*Ct.A—12996.462	0.937	TMD	0.8	Ct.A	0.137	-	-

## Discussion

These studies provide strong evidence that ICWB is predominately remnant woven bone from early life (up to 3 to 4 weeks old), confirm that the ICWB in mice long bones contains calcified cartilage, and show for the first time that whole bone mechanics are affected by the relative composition calcified cartilage. All animals in these studies had ICWB regardless of age or sex. In 12 to 14 week-old mice where early bone (i.e. *in utero* to 3wks old) was fluorescently labeled with DOX the amount and pattern of ICWB closely matched the early bone of the contralateral limb. All animals in both studies had calcified cartilage that were present exclusively within the ICWB, which is consistent with rat studies [7]. Finally, including the percent of the cortical cross-sectional area composed of calcified cartilage with typical strength factors (i.e. Ct.A and TMD) improved the predicative capacity of linear models for maximal force/bending stress.

The long held presumption that ICWB in mouse and rat long bones is from early life is logical. Mouse and rat long bones are highly porous up to 2 months old [8,17], which would suggest a woven bone material. Also, this study as well as a previous rat study found that the absolute amount of WB.A remains fairly constant throughout a rodent’s life. However, the relative amounts of WB.A decline [10]. This suggests that the WB core is laid down during early endochondral or post-natal longitudinal growth and that radial layers of lamellar bone are added around the WB core over time. Indeed, in this study the bone labeled in early life was also encased by bone formed as the animal grew. Moreover, the WB core or EB was not always a contiguous structure around the bone circumference, although the pattern indicated it had been at one point. Nor was it always concentric with the bone cross section. This implies cortical drift during lamellar bone growth. Resorption tended to occur on the anterolateral side while there was more appositional lamellar growth on the posteromedial side (Fig 2B).

An interesting new finding was that the amount of calcified cartilage as a percent of cortical area or woven bone area was higher in older mice than younger mice regardless of sex, although it only reached significance for percent of woven bone. This indicates that the ICWB at the femur mid-shaft in older animals is composed of more calcified cartilage. This could be due to analysis of different absolute bone regions along the bone long axis. Both 6 week and 13 week old bones were analyzed at the femur midpoint. However, Balb/C male and female femora grow an average of 1.5mm or 11% in length between 8 and 16 weeks of age [15]. A majority of the longitudinal growth is likely occurring from the distal growth plate [18]. Thus, the bone at mid-point of the 6 week old bone would probably be proximal of the midpoint in a 13 week old bone. It is possible that there are regional differences in calcified cartilage content along the length of the femur. Tang *et al* showed an uneven distribution with increased calcified cartilage towards the knee joint in both 8-week-old wild-type and MMP13 knockout mouse tibias [19]. This regional difference could potentially explain why there is an increase in cartilage present in the older mice than the younger mice, however, further studies would be necessary.

Most remarkably, the mechanical strength studies found that CC affects whole bone strength and material properties. Percent WB also had some affects. It was the only variable that correlated to post-maximal force energy. Our results indicated that increased percent WB.A positively correlated with ductility. Although, the correlations were very weak (i.e. R^2^ < 0.30). A study of Paget’s disease of the bone in humans, where cortical bone is replaced with a mosaic of lamellar and WB, supports our findings. Despite other changes in material properties, cortical fracture toughness appeared to be maintained due to increased plastic deformation or ductility [4]. On the other hand, a study done on SAMP6 mouse bones, which have decreased collagen organization, found that SAMP6 femora were more brittle than SAMR1 controls [20]. It is ultimately possible that we did not have an adequate range of samples or there were unmeasured confounding variables since the correlation was weak and the results contradict previous mouse studies.

While the mechanics of woven bone have been previously studied, calcified cartilage has not been thoroughly examined. Calcified cartilage percent added small, but significant, predictive power to maximal force and bending stress after Ct.A and/or TMD were considered, where an increase in percent CC correlates to a decrease in both maximal force and bending stress. Maximal force and bending stress are not truly independent measures. Maximal force is normalized by moment of inertia which is dependent on Ct.A. So, it is encouraging that percent CC.A appears in both equations. A previous rat cortical bone study showed that CC is stiffer than the surrounding bone[7]. This could explain the decrease in maximal force/bending stress [7]. However, it should be noted that the range of percent calcified cartilage among these normal samples was not very large (0.87 to 2.31%). Further studies are needed in mouse models where knockout can push physiology outside the normal range to definitively describe the effects of calcified cartilage on whole bone mechanics. In a study of MMP13, a metalloproteinase that plays a role in bone matrix turnover, calcified cartilage percent reached as high as 6% in knockouts; and there were significant changes in bone mechanics despite similar cortical area and tissue mineral density [19]. However, the calcified cartilage was not considered as a strength factor and all differences were attributed to changes in porosity, mineralization heterogeneity, and ICWB area.

The implications of woven bone and calcified cartilage on whole bone strength and bone quality in mouse models are significant. Size and TMD, logically, are the most common factors examined when analyzing bone’s mechanical and material properties. Since the woven bone band is not obvious under standard brightfield imaging nor is calcified cartilage discernible with cone-beam microCT, it is possible that many studies have overlooked their presence and assumed a completely homogenous cortical section, when this is not the case. For most studies, WB.A and CC.A will be secondary factors when size and TMD seem to fully explain the results obtained. However, it could be beneficial to investigate the WB.A and CC.A if studies do not find results explained by size and TMD alone as these secondary factors could be influencing mechanical properties. This could be particularly useful in the case of genetic knockouts where bones have mechanical or material differences where size and/or TMD are similar or cannot fully account for the discrepancy. It should be noted that confidently quantifying WB.A and CC.A may not be possible post-mechanical testing due to tissue damage both from the testing itself and tissue degradation. So it may be prudent to collect additional long bones for this purpose at the time of all other tissue harvests.

Our studies’ caveats should be considered when applying the results to future experiments. One limitation was that only Balb/C mice were used to examine the ICWB and CC changes during aging and effects on whole bone strength. As different mouse strains have distinct bone geometries, mineral densities, and other properties, it is possible that our findings are not relevant to other strains. However, previous studies on various mouse and rat strains have alluded to an intracortical disorganized collagen band [7–11,17,19,21,22]. Also, the mice used for the woven bone origin studies were on a black 6 background and had the same band. We therefore feel confident that most of our findings are strain independent. In addition, only two relatively young ages were analyzed. As older ages were not studied, it is unknown whether the ICWB or CC would remain at older ages of mice nor if the ICWB at the femur mid-shaft is still composed of early bone. However, previous studies in much older animals (up to 12 or 24 months) have touched on the presence of an ICWB, so it is highly likely that ICWB endures throughout a rodent’s life. Furthermore, the use of only two ages may have clustered some data into clouds rather than a regular distribution within the ranges, which can artificially affect linear regression. However, the inclusion of both sexes should have ameliorated this to a degree by providing 3 to 4 clusters for most variables (S1 Table). Finally, our linear models did not include other parameters that can affect whole bone strength and bone quality such as porosity or collagen cross-linking. Both were outside of the scope of these studies.

To conclude, we have shown that mouse bones contain an intracortical band of remnant woven bone from early life (i.e. *in utero* to 3 weeks of age) which contains calcified cartilage. Calcified cartilage affects whole bone mechanics by decreasing maximal force and bending stress. Although, the effects are much weaker than size or TMD. Size and/or TMD, as expected, were the sole or dominating determinates of whole bone strength. Younger bone is composed of more woven bone at the mid-shaft than older bone, while older bone contains higher percent calcified cartilage. There is no significant difference in either woven bone or calcified cartilage composition between males and females. The implications of these results for mechanical tests on mouse cortical bone are significant, and we encourage more studies to look into WB.A and CC.A when conducting phenotypic analysis for mechanical tests, especially if bone size or TMD do not explain discrepancies in mechanical strength.

## Supporting Information

S1 Table Summary of Mechanical Testing Data. Group averages for mechanical testing data.
(XLSX)

S2 Table Linear Regression Data. Data used to create linear regressions.
(XLSX)
